# Expression of CD146 and Regenerative Cytokines by Human Placenta-Derived Mesenchymal Stromal Cells upon Expansion in Different GMP-Compliant Media

**DOI:** 10.1155/2021/6662201

**Published:** 2021-04-02

**Authors:** Frederik Peissert, Hannah D. E. Graf, Bettina Müller, Tanja Abruzzese, Harald Abele, Wilhelm K. Aicher

**Affiliations:** ^1^Department of Urology, University of Tübingen Hospital, Tübingen, Germany; ^2^Department of Gynaecology and Obstetrics, University of Tübingen Hospital, Tübingen, Germany

## Abstract

Mesenchymal stromal cells (MSCs) have been successfully employed in clinical applications. In most studies, autologous MSCs from the bone marrow (bmMSCs) were used, and others employed autologous adipose tissue-derived stromal cells (ADSCs). Recently, clinical feasibility studies provided evidence that MSCs from human term placenta (pMSCs) can be used for homologous therapy facilitating access to regenerative cells in emergency situations, when autologous cells are not available or not suitable. We therefore investigated the expression of MSC stemness marker CD146 and the expression of neuro- and myoregenerative cytokines by human pMSCs after expansion in three different media compliant with good manufacturing protocols (GMP) in comparison to pMSCs expanded in a commercial MSC expansion media. To replace xenobiotic serum in the GMP-compliant media employed in this study, either human serum, human serum plus platelet lysate (PLL), or human plasma plus PLL was used. We report that enrichment of media with PLL accelerates pMSC proliferation but reduces the expression of the stemness marker CD146 significantly, while PLL deprivation enhanced the CD146 expression. In contrast, the reduced expression of CD146 by PLL deprivation was not observed on bmMSCs. The expression of the cytokines investigated was not modulated significantly by PLL. We conclude that accelerated expansion of pMSCs in GMP-compliant media enriched by PLL reduces the expression of stemness marker CD146, but does not influence the expression of neuro- and myoregenerative cytokines.

## 1. Introduction

CD146 is a 113 kDa transmembrane glycoprotein mainly expressed on endothelial cells. Knockdown of CD146 reduced endothelial cell migration and proliferation [1]. It is involved in Ca-dependent adhesion and in outside-in signaling addressing the kinases FYN and FAK as well as the adaptor molecule paxillin [2]. CD146 (alias MCAM, MUC18, and others) is also expressed on melanoma and leukemia cells and considered as an important marker for angiogenesis and cancer [3]. More recently, numerous studies discussed the expression of CD146 on mesenchymal stromal cells (MSCs) from the bone marrow (bmMSCs), adipose tissue (ADSCs), and other sources in the context of bone regeneration [4, 5], vascular commitment [6], proangiogenic activity [7], functions in the bone marrow itself [8, 9], modulation of inflammation [10–12], cellular senescence [13], and in various other aspects [3]. CD146 on bmMSCs is therefore regarded as a marker for multipotent juvenile proliferative progenitor cells [13–16]. Upon expansion of bmMSCs in medium complemented with 10% bovine serum, TGF*β*1 enhanced the expression of CD146 but retarded proliferation, while FGF2 (=bFGF) reduced its expression and activated proliferation [6]. In contrast to bmMSCs and ADSCs, regulation of CD146 on human term placenta-derived MSC-like cells (pMSCs) is less explored. Previously, we reported that the low expression of CD146 on pMSCs correlated with a reduced osteogenic differentiation potential [17] and with significant differences in expression of the osteogenic differentiation factor Runx2 [18, 19]. In addition, we noted that the expression of CD146 on pMSCs, but not on bmMSCs, was in part regulated by the cell culture conditions [20]. As CD146 on MSCs contributes to many important pathways, we now investigate the regulation of this cell surface molecule on pMSCs in more detail. In addition, pMSCs have been used recently in a clinical trial as allogenic cells [21]. In clinical situations, the production of cytokines by the MSCs employed is key for their regenerative potential [22], and MSCs have been utilized to facilitate wound healing [21, 23], modulate immunity and inflammation [10], and regulate many other pathological parameters [24, 25]. We therefore investigated the effects of different GMP-compliant media on the CD146 expression and expression of neuro- and myoregenerative cytokines in comparison to cell culture in a standardized commercial MSC expansion medium.

## 2. Materials and Methods

### 2.1. Isolation and Expansion of MSCs

MSCs were isolated either from the surgical waste of the human bone marrow of patients undergoing prosthesis surgery (*n* = 4, mean 67 years of age), from liposuction tissue of volunteers (*n* =3), or from human term placenta from healthy mothers after cesarian deliveries (*n* = 9, mean 34 years of age) as published earlier [17, 26–28]. Briefly, the bone marrow was diluted with PBS, and mononuclear cells were separated by density gradient centrifugation (470 × g, 20°C, 20 min., Ficoll-Paque; GE Healthcare), washed, and seeded in expansion medium as described below. For isolation of pMSCs and ADSCs, the tissue was cut in fragments and digested by proteolysis (750 U/mL collagenase, Sigma-Aldrich; 250 mg/mL Dispase, Roche; 37°C, 60 min.) in moderate motion. Proteolysis was stopped by the addition of serum. Cells were washed and separated by Ficoll-Paque gradient centrifugation, washed again, and cultured. To obtain fetal (fpMSC) versus maternal (mpMSCs) pMSCs specifically, the placenta was sliced to separate the endometrial from the amniotic parts. The maternal and fetal parts were then processed individually as described above.

The cells were expanded either in a commercial MSC expansion medium (MSCGM, Lonza, = LM) or in low glucose DMEM medium (Sigma-Aldrich) complemented by GMP-compliant components: human serum, human serum plus human platelet lysate (PLL), or human plasma plus PLL (Table 1), antibiotics (pen/strep, Lonza), and L-glutamine (Lonza) and buffered by HEPES (Lonza) as described [28, 29]. Pooled batches of human serum and PLL were obtained from the Institute of Clinical and Experimental Transfusion Medicine at University of Tuebingen Hospital. The PLL was prepared as described recently [30]. Human plasma was obtained from TCS Biosciences (Botolph Claydon, Buckingham MK182LR, UK). Unless otherwise stated, for the PLL induction experiments, cells were expanded to their second passage in a medium w/o PLL, harvested, and split in two sets. One set was continued for 1, 3, or 7 days in the same medium. The other set was continued with medium enriched by PLL (Table 1). Comparably, for PLL deprivation experiments, cells were expanded in a given medium in presences of 5% PLL, harvested, and split in two sets. One set was continued for 1, 3, or 7 days in medium enriched with 5% PLL. The other set was continued with growth medium without PLL (Table 1). The data were normalized to the respective controls or to cells expanded in LM (=100%). All stromal cell populations were investigated to meet the MSC inclusion criteria defined for bmMSCs [26].

To determine cell proliferation, MSCs were expanded to their 2^nd^ passage, harvested, and counted, and 3E05 cells per flasks were seeded on day *x*. When reaching 80% of confluence on day *x* + *t*, MSCs were harvested and counted, and the proliferation was computed using [log_10_(cell count day *x* + *t*) − log_10_(3E05)]/log10 (2) = population doublings in *t* days.

### 2.2. Analysis of Steady-State mRNA Amounts

The expression of mRNA transcripts was explored by quantitative RT-PCR (qRT-PCR) as described [17]. In brief, cells were harvested and washed, and RNA was isolated by aid of the RNeasy kit (Qiagen). The yield was determined by photometry (NanoPhotometer, IMPLEN), and cDNA was generated from 1 *μ*g total RNA using oligo (dT) priming (Advantage RT-for-PCR kit, TaKaRa-Clontech). For quantification of cDNAs, a polymerase chain reaction (PCR) was performed using the LightCycler 480 SYBR I Master Kit and hardware as requested by the provider (Roche) using the following temperature settings: denaturation 94°C, annealing 60°C, and primer extension at 72°C. Primers used for CD146, glycerinaldehyde 3-phosphate dehydrogenase (GAPDH), and peptidylprolyl isomerase A gamma (PPIA*γ*) qRT-PCR are listed in Table 2. The amounts of the target gene CD146 were computed employing the two internal standard transcripts GAPDH and PPIA*γ* as described by Roche software [31]. For detection of transcripts encoding cytokines, commercial primers were used (Qiagen).

### 2.3. Detection of Proteins by Immunoblotting

The CD146 protein was detected in cell lysates after SDS-polyarcrylamide gel electrophoresis (SDS-PAGE) and immune blotting [32]. In brief, 1E06 cells were harvested by mild proteolysis employing accutase (Roche) and homogenized in 1 mL lysis buffer (150 mM NaCl, 10 mM Tris-HCl pH 7.4; 1% Triton X-100; 0.1% SDS, 1 mM PMSF) [32]. The protein yield was determined by colorimetry as requested by the supplier (DC Protein Assay Kit II, BioRad). 50 *μ*g of total protein extract was separated by SDS-PAGE and transferred to nylon membranes. The membranes were blocked by 5% dry milk in PBS enriched by 1% Tween20 [32]. CD146 was detected by antibody (1 : 1000 in PBS-Tween20, rb-ā-huCD146 mAb, Abcam), followed by detection serum (1 : 300 in PBS-Tween20; HRP-labelled gt-ā-rb serum, Dako) and recorded by chemiluminescence (western sure premium; Licor) in a scanner (C-Digit, Licor). After stripping the membrane, detection of the *β*-actin expression served as control. Signal intensities of CD146 and *β*-actin were calculated by ImageStudio (https://www.licor.com/bio/software) and NIH-Image (https://imagej.nih.gov/nih-image/) software.

### 2.4. Detection of Cell Surface Proteins by Flow Cytometry

The expression of cell surface markers on MSCs was investigated by flow cytometry as described [17]. In brief, cells were harvested by mild proteolysis (Accutase), washed, counted, and incubated with fluorochrome-labeled monoclonal antibodies to CD14, CD73 (BD Pharmingen), CD34, CD45 (BioLegend), CD90, CD146 (R&D Systems), or CD105 (AbD Serotec), as requested by the suppliers. After incubation, cells were washed and resuspended in PFEA buffer (PBS, 2% fetal bovine serum, 2 mM EDTA, 0.01% sodium azide) for flow cytometry employing a flow cytometer (LSR II, BD Bioscience) [17]. Cells incubated with an isotype antibody and COMP beads (BD) served as controls. The mean fluorescence intensities (MFI) of staining and flow cytometry data were computed by FACS Diva software (BD Bioscience) and then processed by FlowJo 10.3.0 (Treestar) software to prepare the figures.

### 2.5. Statistics

The experimental data were processed by a spread sheet app (Excel, Microsoft). Statistical significance was computed by a two-sided unbiased *t*-test. *P* values smaller than 0.05 were considered significant.

## 3. Results

### 3.1. Expression of CD146 on bmMSCs versus pMSCs after Expansion in Different GMP-Compliant Media

**Figure 1:**
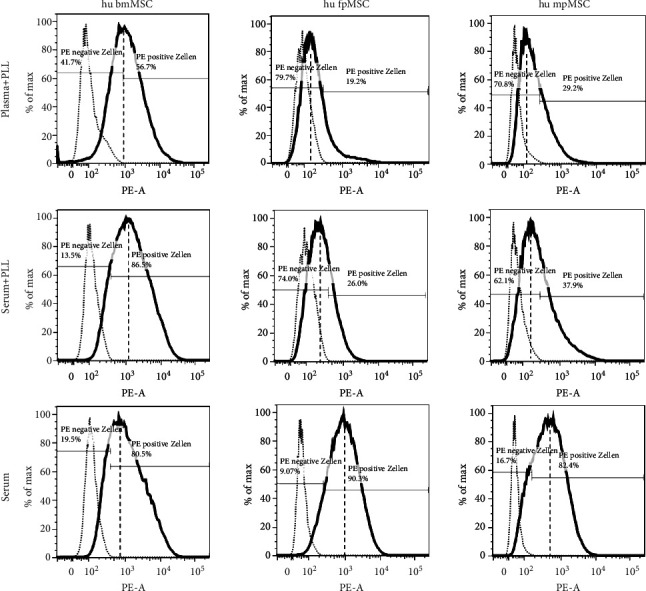
The expression of CD146 on bmMSCs and pMSCs. The bmMSCs, fpMSCs, or mpMSCs were expanded in GMP-compliant media complemented with plasma plus PLL, serum plus PLL, or serum as indicated. The expression of CD146 was determined by flow cytometry. While the CD146 expression did not differ among bmMSCs expanded in the different media, expansion of fpMSCs and mpMSCs in media complemented by PLL expressed less CD146 when compared to the same cells expanded in media complemented with serum but without PLL.

In the first set of analyses, bmMSCs and pMSCs from either the fetal (fpMSCs) or maternal (mpMSCs) parts of the term placenta were expanded a priori in three different GMP-compliant expansion media complemented with either plasma plus PLL, serum plus PLL, or serum. The expression of CD146 was enumerated by flow cytometry (Figure 1). The expression of CD146 was not different on bmMSCs expanded in media containing human serum or plasma enriched by PLL in comparison to media complemented by serum only, and the mean fluorescence intensities (MFI) of CD146 were recorded in the range of 1900 to 2500 (Figure 1). In contrast, for both fpMSCs as well as mpMSCs, PLL enriched expansion medium reduced the CD146 expression. In media complemented by serum only, fpMSCs as well as mpMSCs expressed more CD146 than after expansion in media complemented by PLL. The fpMSCs expanded medium complemented with serum only reached CD146 levels measured on bmMSCs in the same medium (Figure 1). In a separate study, the CD146 expression was explored in pMSCs from 4 additional donors expanded either in LM or in GMP-compliant DMEM medium complemented by serum and enriched by PLL. On pMSCs expanded in LM, the MFI of CD146 was 3626 ± 1110, and 75% of cells were gated positive. In contrast, in GMP-compliant DMEM media enriched by serum plus PLL, the MFI of CD146 (780 ± 521, *n* = 4 each; *P* < 0.004) and the percentage of cells gated positive (59%; *P* < 0.03) were significantly lower (not shown). This corroborated our earlier studies [17, 20]. As the addition of PLL to the expansion media caused a robust reduction of CD146 on pMSCs, we investigated the influence of PLL on THE CD146 expression by human pMSCs in more detail.

### 3.2. Effect of PLL on the Expression of CD146 on pMSCs

**Figure 2:**
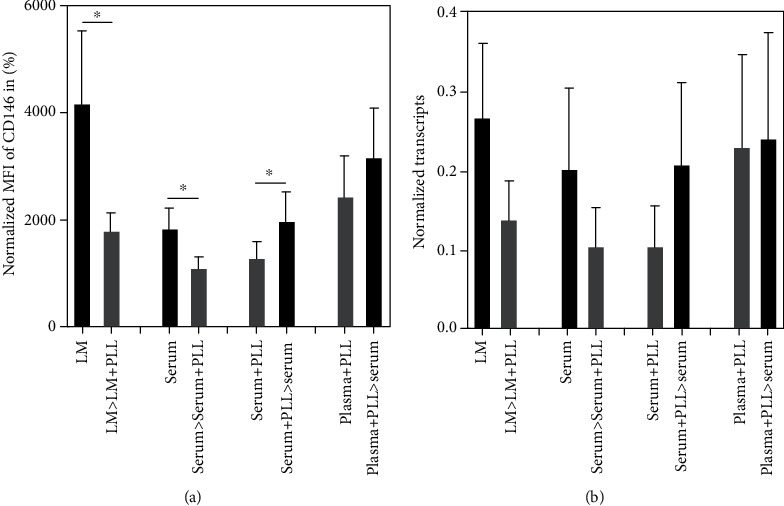
Effect of PLL on the CD146 expression. The pMSCs were expanded to their second passage in medium as indicated, split, and expanded further for 7 days in the same medium (i.e., LM, serum, serum+PLL, plasma+PLL) or in medium either enriched (>LM + PLL, >serum+PLL) or deprived from PLL (>serum, >serum). (a) The average of CD146 mean fluorescent intensities (MFI) dropped in all experiments when pMSCs were further cultured in medium complemented with PLL (grey bars), while it increased, when pMSCs were further cultured in medium deprived from PLL (black bars). The bars show the mean MFI ± standard deviation from pMSCs of 5 donors. (b) Messenger RNA amounts were enumerated by qRT-PCR in pMSCs expanded in medium either complemented with PLL (grey bars) in comparison to medium without PLL (black bars). In all cases investigated, addition of PLL reduced CD146 transcript amounts, while reduction of PLL from expansion in serum+PLL to serum only caused an increase in CD146 transcripts. The change from plasma+PLL to serum had no effect on CD146 transcript amounts. The bars show the mean normalized transcript amounts from pMSCs of 3 donors.

In the second set of experiments, we compared the effects of PLL on the CD146 expression in different media combinations (Figure 2). To this end, the pMSCs were expanded ex vivo to their second passage in vitro in either LM or in GMP-compliant media enriched with 10% human serum only. Of note, human MSCs did not grow in medium complemented with plasma only (not shown). Then, cultures were split, and one set was maintained for 7 days in the same medium (i.e., LM or DMEM+10% serum). The other set was expanded for 7 days in presence of PLL, i.e., in LM+5% PLL or in DMEM+5% serum+5% PLL, respectively. The expression of CD 146 was explored by flow cytometry and by qRT-PCR (Figure 2). The expression of the CD146 expression was significantly reduced on the cell surfaces upon addition PLL to pMSCs expanded in LM (*n* = 5, *P* < 0.05) or in DMEM+serum (*P* < 0.04; Figure 2(a)). The same trend was observed on the transcript levels (*n* = 3, Figure 2(b)).

In contrast, when pMSCs were initially expanded ex vivo in DMEM media enriched with serum plus PLL and then deprived of PLL by further expansion of the cells in DMEM media enriched with serum only, the MFI of the CD146 expression increased significantly (*n* = 5, *P* < 0.03; Figure 2(a)). An increase in CD146 transcript amounts was also seen in cells changed from DMEM+serum+PLL to DMEM+serum only (*n* = 3, Figure 2(b)). No increase was seen when cells were changed from DMEM+plasma+PLL to DMEM+serum only (Figure 2). Of note, the overall CD146 expression levels were different in cells initially expanded in DMEM+serum (MFI 2072 or 1697) when compared to pMSCs expanded initially in DMEM+plasma+PLL followed by PLL deprivation and further expansion in DMEM+serum (MFI 5720). The expression of CD73, CD90, and SUSD2 on pMSCs cultured in the different media was analyzed by flow cytometry as well, but remarkable PLL-dependent differences were not noted (not shown).

### 3.3. Kinetics of Regulation of CD146 by PLL

**Figure 3:**
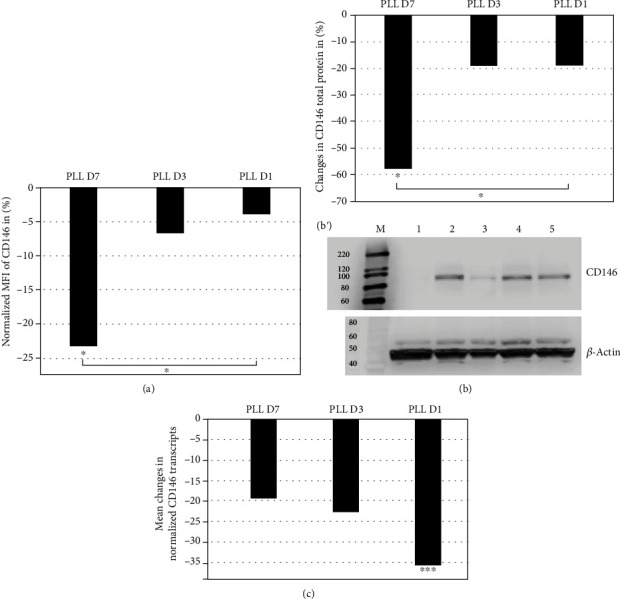
Time line of PLL effects on the expression of CDC146. The pMSC from 4 donors was expanded in LM to their second passage and further expanded in LM+5% (vol/vol) PLL. Controls were incubated in LM. (a) The expression of CD146 on cells surfaces was investigated by flow cytometry. Seven days after addition of PLL, the MFI of the CD146 expression dropped significantly in comparison to pMSCs in LM and in comparison to cells 1 day after addition of PLL (*P* < 0.05 each). (b) The expression of the total CD146 protein was investigated by immunoblot. Seven days after addition of the PLL total protein of the CD146 expression dropped significantly in comparison to pMSCs in LM and in comparison to cells 1 day after addition of PLL (*P* < 0.05 each). (B') A representative immunoblot is shown in the lower right: CD146 is not detected in control cells (lane 1), but in pMSCs expanded for 7 days in LM (lane 2), almost not in pMSCs expanded for 7 days in LM + PLL (lane 3). CD146 is detected in pMSCs expanded for 72 hours (lane 4) or 24 hours (lane 5) in LM + PLL. Detection of ß-actin served as loading control. Size markers are shown in lanes M. The molecular weight of proteins separated by SDS-PAGE is noted on each blot. (c) The expression of transcripts encoding CD146 was enumerated by RT-PCR. After 24 hours of incubation in LM + PLL, a significant drop in CD146 transcripts was noted in comparison to pMSCs in LM (*P* < 0.005). After 3 and 7 days, CD146 encoding transcript increased somewhat but stayed well below the levels recorded in pMSCs in LM.

To investigate the time line of PLL-mediated regulation of CD146, pMSCs were expanded in LM to their second passage and split. PLL (5%) was added for either 1, 3, or 7 days, and the expression of CD146 was recorded on cell surfaces by flow cytometry, by immunoblotting to determine total protein amounts and on the transcriptional level by qRT-PCR (Figure 3). A minor reduction of the CD146 MFI on cell surfaces was observed as early as 1 and 3 days after PLL addition by flow cytometry. However, after 7 days, a significant drop in CD146 staining was observed (*n* = 4, *P* < 0.05, Figure 3(a)). A significant drop of the total CD146 protein was recorded 7 days after PLL addition as well by immunoblotting (*n* = 4, *P* > 0.05, Figure 3(b)). Regulation of CD146 on the transcript level occurred with distinct kinetics. A significant drop of CD146 encoding transcripts was enumerated as early as one day after PLL addition (*n* = 4, *P* < 0.005). However, transcript amounts increased within the following days. The steady-state transcript amounts encoding CD146 remained below the levels recorded in pMSCs prior to the addition of PLL (Figure 3(c)). We conclude that PLL modulates the CD146 expression in part on the transcriptional level.

### 3.4. Effect of PLL on Proliferation and Cytokine Expression of pMSCs

**Figure 4:**
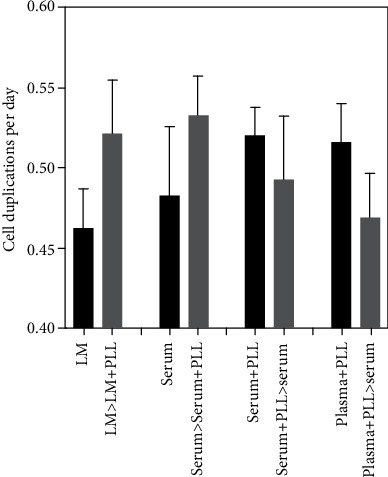
Effect of PLL on proliferation of pMSCs. The pMSCs from 5 donors were expanded either in LM, medium complemented by serum, serum+PLL, or plasma+PLL and further cultured for 2 consecutive passages in medium enriched (grey bars) with or deprived from PLL (black bars) as indicated. Addition of PLL to expansion media increased cell proliferation in both cases (left side), while PLL deprivation reduced proliferation in the other two cases (right side).

As the expression of CD146 on MSCs was associated with their proliferation capacity, we enumerated the mitotic activity of pMSCs in media enriched with PLL in comparison to the same media without PLL. In all combinations investigated, starvation of PLL retarded cell proliferation, while the addition of PLL accelerated the proliferation of pMSCs (Figure 4). This corroborated our previous study employing human bmMSCs [28]. We conclude that PLL enhances the proliferation of human pMSCs in GMP-compliant media required for (pre-) clinical studies, but it reduces the expression of CD146. Thus, PLL may indirectly have an impact on the “stemness” of human pMSCs.

**Figure 5:**
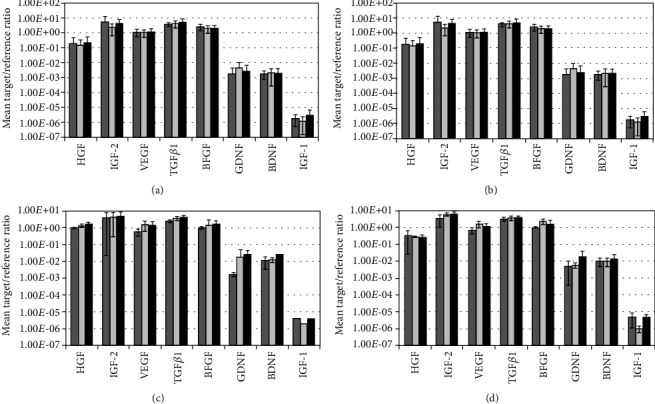
The expression of cytokines in different MSCs in different GMP-compliant media. The bmMSCs (a), ADSCs (b), fpMSCs (c), and mpMSCs (d) were expanded ex vivo in 3 different GMP-compliant media (serum = dark grey bars, serum + PLL = light grey bars, plasma + PLL = black bars). Transcripts encoding HGF, IGF-2, VEFG, TGF-*β*1, BFGF, GDNF, BDNF, and IGF-1 were enumerated by qRT-PCR. The graphics show the mean target gene to reference gene ratios from *n* ≥ 3 individual MSC expansions and analyses.

Next, the transcript levels encoding neuro- and myoregenerative cytokines were explored in bmMSCs, ADSCs, fpMSC, and mpMSCs, after expansion in GMP-compliant media (Figure 5). Major differences between bmMSCs, ADSCs, and pMSCs were not observed nor prominent effects of PLL on the regulation of an individual cytokine. Expression of factors involved in myogenesis and vascularization, hepatocyte growth factor (HGF), insulin-like growth factor-2 (IGF-2), vascular endothelial growth factor (VEFG), transforming growth factor beta 1 (TGF-*β*1), and basic fibroblast growth factor (BFGF), respectively, reached prominent transcript levels. Transcripts encoding factors involved in neuroregeneration, glial cell-derived neurotrophic factor (GDNF), and brain-derived neurotrophic factor (BDNF) were enumerated about 100-fold lower, and insulin-like growth factor-1 (IGF-1) was even 3 to 5 logs below the other factors (Figure 5). As serum and PLL are rich in cytokines, cytokine release by cells was not explored in culture supernatants.

## 4. Discussion

The expression of the adhesion molecule CD146 on pMSCs is modulated by factors contained in PLL, while in bmMSCs, PLL did not modulate the expression of this “stemness marker.” Therefore, the composition of the media employed to produce pMSCs for (pre-) clinical purposes is more critical when compared to the production of bmMSCs. The elevated expression of CD146 on MSCs was associated with their differentiation capacity, proliferation, and telomerase activity [13]. In some cells, the expression of CD146 was activated by TNF-*α*, in others by endothelin-1, IL-1, IL-13, TGF-*β*, or NGF [3]. PLL contains PDGF's, IGF-1, TGF-*β*s, and other cytokines [33], indicating that these factors can contribute to the regulation of CD146. All media combinations investigated in our study documented that PLL reduced the expression of the stem cell marker CD146. This suggested that factors contained in PLL—but probably not TNF-*α*—regulated CD146 on pMSCs.

Glucose and intracellular Ca^2+^ ions were shown to activate the CD146 promotor via elevated cAMP, thus again promoting the CD146 expression [3]. However, the PLL effects were observed independently in all media investigated here. In human plasma (119.4 ± 9.9 mg/dL) vs. serum (108.5 ± 6.5 mg/dL), comparable Ca^2+^ concentrations were reported [34], suggesting that the Ca^2+^ signaling was not mainly involved in CD146 regulation observed here. Comparably, glucose concentrations in DMEM basic media (100 mg/dL), human blood (80–120 mg/dL), plasma (70–130 mg/dL), or serum (25–250 mg/dL) are within the same range. We therefore conclude that glucose does not play a major role for theCD146 expression on pMSCs. However, plasma contains fibrinogen, and soluble fibrinogen may activate cells via integrin ß1- or *β*2-integrins and NF-*κ*B signaling [35, 36]. Fibrinogen modulated MSC proliferation and in vitro differentiation. Fibrin-modulated gene expression in MSCs was reported [37, 38]. Therefore, the content of fibrinogen or fibrin in MSC expansion media may modulate the CD146 expression. Moreover, dependent of the batch and preparation protocol used, PLL contains different amounts of bFGF [33]. bFGF enhanced bmMSC proliferation and suppressed CD146 expression [6, 8, 16]. The effects observed in this study are in line with the effects reported for bFGF. We therefore conclude that bFGF may contribute to the regulation of CD146 on pMSCs in a different way when compared to bmMSCs. The prominent bFGF expression was recorded in pMSCs upon expansion in the same GMP-compliant media as employed here (not shown).

Nowadays, most strategies employing MSCs in the context of preclinical or clinical studies see such cells as sources for enhanced production of regenerative factors and for immunomodulation [12, 22, 39, 40]. However, species, tissue, and donor-dependent differences of cytokine production were observed. In mice, activated CD146^neg^ MSCs expressed more IL-6 when compared with CD146^pos^ cells, while the secretion of TGF-*β*1 remained unchanged upon activation [12]. CD146^neg^ MSCs caused a significant activation of Th17 T-leukocytes and augmented an experimental inflammation in an animal model. In contrast, CD146^pos^ cells ameliorated the inflammation [12]. Knockdown of the CD318 expression in CD318^pos^/CD146^neg^ stromal cells caused an elevated production of G-CSF, IL-1*β*, and IL-8 and others, while IL-6 and VEGFs were not changed [41]. Enrichment of CD146^low^ cells by expansion of pMSC in PLL media may therefore in part reduce their regenerative potential in clinical situations. This may be relevant when MSC differentiation comes into account. However, many current studies suggest that the main action of MSCs in vivo is associated with their release of factors promoting wound healing, modulating inflammation, and facilitating vascularization [22, 40]. Our studies suggest that the mRNA expression of key cytokines involved in the regulation of wound healing, muscle and nerve regeneration, and in angiogenesis did not significantly differ when bmMSCs, ADSCs, fpMSCs, and mpMSCs were expanded in presence of absence of PLL [42].

As PLL is very rich in PDGFs, IGF-1,and TGFs [33], we did not perform analyses of cytokine release by MSCs in PLL media. We considered that PLL and culture conditions would cause a major bias in such studies. In addition, the investigation of intracellular cytokine amounts is beyond the focus of this study and therefore was not explored. Another limitation of the study is the fact that bmMSCS were obtained from elderly donors while placenta is obtained from comparably young mothers. Therefore, an exact match of donor gender and age is of course impossible. Moreover, changes in cellular appearance—as determined by microscopy—seem to depend more on time of in vitro culture and population doublings and to a lesser degree on the composition of the media investigated here. Human term placenta comes here with a clear advantage.

MSCs can be isolated from human placenta with only little ethical issues and from a rather large and well vascularized tissue. This yields many cells from one sample and requires less in vitro expansion to generate the cells needed. The placenta contains “young cells” with long telomers facilitating extended in vitro expansion without an elevated risk of replicative senescence [19]. Finally, their isolation yields no disadvantage to the donor.

Bone marrow-derived MSCs characterized by elevated levels of “stemness marker” CD146 express in vitro cytokine patterns not significantly different from pMSCs which express significantly less CD146. As bmMSCs tend to undergo osteogenesis in vivo with much higher efficacy when compared to pMSCs [17–20, 43], pMSCs may be a remarkable replacement for bmMSCs in different applications and cell therapies, including a regimen for local continued release of growth factors.

## 5. Conclusions

Enriching GMP-compliant media by PLL activated the proliferation of pMSCs. In a clinical situation, this facilitates faster expansion of pMSCs to the numbers needed for the application. Although expression of the stem cell marker CD146 is reduced by PLL in pMSCs in all media combinations investigated here, the expression of the cytokines explored here was not altered significantly. As pMSCs have been approved for feasibility studies with allogenic recipients, pMSCs may become an interesting candidate cells for treatment of a wide variety of disorders.

## Figures and Tables

**Table 1:** Composition of GMP-compliant MSC expansion media.

Medium	Serum	Serum+PLL	Plasma+PLL
Pooled human serum	10%	5%	0
Pooled human plasma	0		5%
Pooled human PLL	0	5%	5%

**Table 2:** Primers for qRT-PCR.

Gene	Primer direction	Sequence (5′→3′)	NCBI Acc. #
CD146	Forward	GAAGTCACCGTCCCTGTTTTC	NM 006500.3
	Reverse	CCCCGTTGTCGTTGGTTGT	dto.
GAPDH	Forward	GAGTCAACGGATTTGGTCGT	NM 002046.7
	Reverse	TTGATTTTGGAGGGATCTCG	dto.
PPIA*γ*	Forward	TTCATCTGCACTGCCAAGAC	NM 021130.5
	Reverse	TCGAGTTGTCCACAGTCAGC	dto.

## Data Availability

All data included in this study are available from WKA to colleagues in the research on reasonable request.

## Abbreviations

ADSC: Adipose tissue-derived stromal cells, alias atMSC
bmMSC: Bone marrow-derived MSC
FACS: Fluorescence activated cell sorting
fpMSC: Fetal pMSC
GMP: Good manufacturing protocols
LM: Lonza medium, MSC growth medium (MSCGM) from Lonza
mpMSC: Maternal pMSC
MSC: Mesenchymal stromal cell/mesenchymal stem cell
PLL: (Human) platelet lysate
pMSC: (Human term) placenta-derived MSC
SUSD2: adhesion molecule associated with cancer and stem cell marker on bmMSCs.

## Additional Points

*Highlights.* (i) Expansion of pMSCs in media enriched PLL enhances cell expansion but reduces the expression of stemness marker CD146. (ii) Expression of neuro- and myoregenerative cytokines is not modulated by PLL in pMSCs. (iii) Addition of PLL complements GMP-compliant expansion media for accelerated production of pMSCs in (pre-) clinical situations
